# Genomic Transcriptome Benefits and Potential Harms of COVID-19 Vaccines Indicated from Optimized Genomic Biomarkers

**DOI:** 10.3390/vaccines10111774

**Published:** 2022-10-22

**Authors:** Zhengjun Zhang

**Affiliations:** Department of Statistics, School of Computer, Data & Information Sciences, University of Wisconsin, Madison, WI 53706, USA; zjz@stat.wisc.edu

**Keywords:** vaccine response, biological equivalence, competing risks, gene–gene interaction, BNT162b2 vaccine

## Abstract

COVID-19 vaccines can be the tugboats for preventing SARS-CoV-2 infections when they are practical and, more importantly, without adverse effects. However, the reality is that they may result in short-term or long-term impacts on COVID-19-related diseases and even trigger the formation of new variants of SARS-CoV-2. Using published data, we use a set of optimized-performance COVID-19 genomic biomarkers (MND1, CDC6, ZNF282) to study the benefits and adverse effects of the BNT162b2 vaccine. We found that the vaccine lowered the expression values of genes MND1 and CDC6 while heightening the expression values of ZNF282 in individuals who are SARS-CoV-2 naïve, which is expected and satisfies the biological equivalence between the COVID-19 disease and the genomic signature patterns established in the literature. However, we also found that COVID-19-convalescent octogenarians responded reversely. The vaccine heightened the expression values of MND1 and CDC6. In addition, it lowered the expression values of ZNF282. Such adverse effects raise outstanding concerns about whether or not COVID-19-convalescent individuals should take the current vaccine or when they can take it. These findings are new at the genomic level and can provide insights into developing next-generation vaccines, antiviral drugs, and pandemic management guidance.

## 1. Introduction

Since the pandemic started, knowledge of virus structures at protein levels has been obtained, and many informative publications have been supplied [1,2,3]. In the meantime, publications related to SARS-CoV-2 genetics and vaccine effectiveness offered many valuable insights for further research and practice, including pandemic management [4,5,6,7,8,9,10,11,12,13,14,15,16,17,18,19]. However, there have been reports that SARS-CoV-2 variants evade immune response [20,21,22] and that adverse effects have resulted from vaccines [23,24], which has raised concerns about the decreased effectiveness and increased adverse effects of current vaccines. In addition, understanding of COVID-19′s pathological causes, virus formations, and gene–gene interactions is still limited. At the genomic level, Zhang [25,26,27] advanced a powerful new study method to establish a geometry of COVID-19 genome space that sets the biological equivalence between the COVID-19 disease and the three genomic signature patterns and seven subtypes of the disease; see [28,29,30,31] for cancer studies. Such genomic-level advances bring new hope in fighting against the COVID-19 pandemic. 

Antibody responses to SARS-CoV-2 and variants have been commonly applied in evaluating the efficacy of vaccines. We recently identified a set of critical genes that can perfectly or nearly perfectly classify the individuals into their respective groups and further cluster the COVID-19-infected into subgroups. Such high performance led to the identification of reliable biomarkers of COVID-19 disease using blood-sample data [25,26] and of SARS-CoV-2 using NP (nasopharyngeal)/OP (oropharyngeal) swab PCR-sample data [27]. We, in this paper, use the high-performance gene biomarkers to evaluate the genomic benefits and adverse effects of the BNT162b2 vaccine in COVID-19-convalescent octogenarians and SARS-CoV-2-naïve individuals. Here, the genomic benefits mean that the vaccine will increase (decrease) a gene’s expression value if the higher (lower) expression value of this gene will lower an individual’s SARS-CoV-2 infection risk, and adverse effects mean the reversed direction. 

The contributions of this paper are three-fold: (1) signifying genomic (MND1 (meiotic nuclear divisions 1), CDC6 (cell division cycle 6), ZNF282 (zinc finger protein 282) benefits and adverse effects of the COVID-19 vaccine BNT162b2 in two heterogeneous populations; (2) pointing to a new direction of COVID-19 studies, i.e., gene–gene interactions; (3) pointing to a potential target of next-generation vaccines, antiviral drugs, treatments, and management. In (1), we first found that the vaccine lowered the expression values of genes MND1 and CDC6 while heightening the expression values of ZNF282 of individuals who are SARS-CoV-2 naïve, which is as expected and satisfies the biological equivalence between the COVID-19 disease and the genomic signature patterns established in the literature [26]. However, we also found that COVID-19-convalescent octogenarians responded to the vaccine reversely. The vaccine heightened the expression values of MND1 and CDC6. In addition, it lowered the expression values of ZNF282. Such adverse effects raise outstanding concerns about whether or not COVID-19-convalescent individuals should take the current vaccine or when they can take it. 

The remaining part of the paper is organized as follows. First, Section 2 briefly reviews the study methodology. Next, Section 3 reports the data sources, analysis results, and interpretations. Finally, Section 4 concludes the study with discussions. Supplementary Materials contain real data and computer outputs.

## 2. Method

We use two methods to conduct our comparative studies. The first method is a graphical method (popular in biological and medical research), without any statistical computation, which directly plots and demonstrates gene expression level changes over time after vaccinations. Here, the original gene expression level changes represent clinical information, such as antibody responses. Such a direct method reveals the biological and clinical information related to heterogeneous populations and their genomic effects. The second method is to apply the proven method of max-linear competing logistic regression classifier to the classifications of BNT162b2 vaccine responses between COVID-19-convalescent individuals and SARS-CoV-2-naïve individuals. The second method is very different from other classical statistical and modern machine learning methods, e.g., random forest, deep learning, and support vector machine. In addition, the new method has enhanced the interpretability of results, consistency, and robustness, as shown in our earlier work [25,26,27,28,29,30,31] on studies of COVID-19 and the biomarkers of several types of cancers.

The genes selected in this new study were chosen from the genes identified in our earlier work [25,26,27], which led to perfect or nearly perfect performance. As a result, they can be used as reliable biomarkers, which will be further justified in this new comparative study. 

## 3. Data Descriptions, Results, and Interpretations

### 3.1. The Data and Our Earlier Results

The data we used in this comparative study of the BNT162b2 vaccine’s gene expression responses are publicly available as GSE190747 [32] and the outcome data in our earlier work [25,26,27] using GSE157103 [33], GSE152418 [34], https://github.com/czbiohub/covid19-transcriptomics-pathogenesis-diagnostics-results(accessed on 26 December 2021) [35], and GSE152075 [36]. 

The GSE190747 data contain (1) 16 female COVID-19-convalescent octogenarians and their gene expression values collected at days 0, 1, and 7; and (2) 14 COVID-19-naïve individuals (ages from 26 to 72) and their gene expression values collected at days 0, 10, 11, 34, 35, 40, 41, 48. 

We, here, briefly report our earlier results [26] that established the existence of genomic signature patterns and COVID-19 subtypes and the mathematical and biological equivalence of the disease and the signature patterns. The work used max-linear competing logistic regression models to establish component classifiers CF-i and the combined max classifier CFmax. The following Table 1 and Table 2 appeared in our earlier work [26]. 

For the GSE157103 data, we also found that a combination of CDC6 and ZNF282 can lead to 97.62% accuracy (98% sensitivity, 96.15% specificity), with the following classifier: 1.7615 + 6.8226 × CDC6 −1.1556 × ZNF282; a combination of CDC6, ZNF282, and CEP72 (centrosomal protein 72) can lead to 98.41% accuracy (99% sensitivity, 96.15% specificity), with the following additional classifier: −1.9944 + 7.4106 × CDC6 −7.1401 × CEP72.

As the pandemic is now dominated by Omicron variants, especially BA.5, linking the genes identified earlier for other variants to Omicron variants will provide better genomic knowledge of COVID-19 diseases. We found that the genes identified from GSE157103 and GSE152418 again led to 100% accuracy with a SARS-CoV-2 Omicron variant BA.1 cohort study GSE201530 [37]. The following new Table 3 reports the outcomes.

The data from GSE201530 contains four types: unvaccinated/no prior infection, vaccinated/no prior infection, unvaccinated/prior infection, and vaccinated/prior infection. Comparing Table 3 with Table 1 and Table 2, we can see different patterns in fitted coefficients associated with the chosen genes, which is not surprising, as COVID-19 patients in two previous cohorts (GSE157103 and GSE152418) were first-time infections and had no vaccinations, i.e., GSE157103 and GSE152418 have different group comparisons to GSE201530. An essential feature in Table 1 and Table 2 is that the signs and strengths of fitted coefficients are interpretable, i.e., they tell how the expression level changes of the biomarker genes affect the risk of COVID-19 infection and their functional effects. However, the genes identified from our earlier work still lead to 100% accuracy in Table 3, which shows that these genes contain information related to SARS-CoV-2 variants, including Omicron BA.1, and likely BA.5 (once the data are available to check).

For the NP/OP swab PCR-sample data—https://github.com/czbiohub/covid19-transcriptomics-pathogenesis-diagnostics-results [35]—the following Table 4 appeared in our earlier work [27]. 

As discussed in our earlier work [27], genes in Table 4 and Table 5 and their transcriptional response and functional effects on SARS-CoV-2 and genes in Table 1, Table 2 and Table 3 and their functional signature patterns to COVID-19 antibodies are significantly different, which can be interpreted as the former being the point of a phenomenon, and the latter being the essence of the disease. Such significant findings can help explore the causal and pathological clues between SARS-CoV-2 and COVID-19 disease and fight against the disease with more targeted vaccines, antiviral drugs, and therapies. Putting Table 1, Table 2, Table 3, Table 4 and Table 5 together serves as a starting point for our new comparative vaccine efficacy study in the subsequent sections. 

Given the perfect performance of genes (ABCB6 (ATP Binding Cassette Subfamily B Member 6 (Langereis Blood Group)) KIAA1614, MND1, SMG1 (nonsense-mediated mRNA decay associated PI3K related kinase), and RIPK3 (Receptor Interacting Serine/Threonine Kinase 3)) in Table 1, Table 2 and Table 3 using blood-sample data and the nearly perfect performance of genes CDC6, ZNF282, and CEP72, these genes certainly can be used as reliable biomarkers for COVID-19 diseases (blood samples). On the other hand, ATP6V1B2 (ATPase H+ Transporting V1 Subunit B2) and IFI27 (Interferon Alpha Inducible Protein 27) have central roles in SARS-CoV-2 heterogeneous populations, which was discussed in our earlier work [27] and is further confirmed in the new Table 5 using NP/OP swab PCR samples. Therefore, considering the functions of these genes discussed in our earlier work [26,27], we focus on the genes MND1, SMG1, CDC6 (cell division cycle 6), ZNF282, CEP72, ATP6V1B2, and IFI27 in this study using the data of BNT162b2 vaccine efficacy [32]. 

### 3.2. The Clinic Evidence Directly Observed Using Graphical Approach and Results

**Figure 1:**
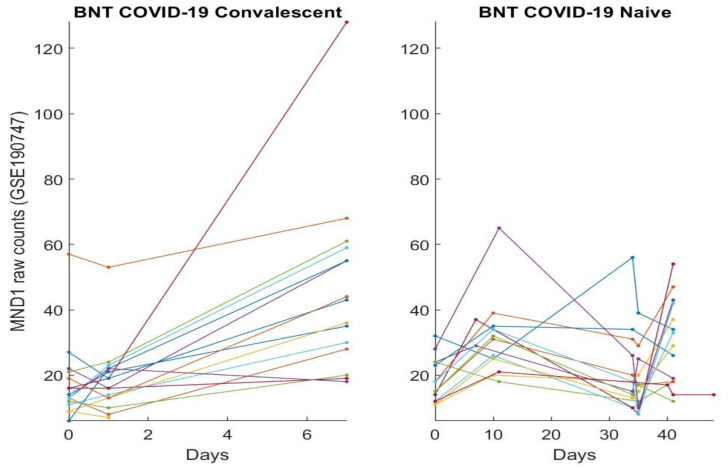
MND1 expression responses to BNT162b2 vaccines: The left panel is for COVID-19-recovered octogenarians; the right panel is for COVID-19-naïve individuals. Each color line corresponds to one patient’s records.

**Figure 2:**
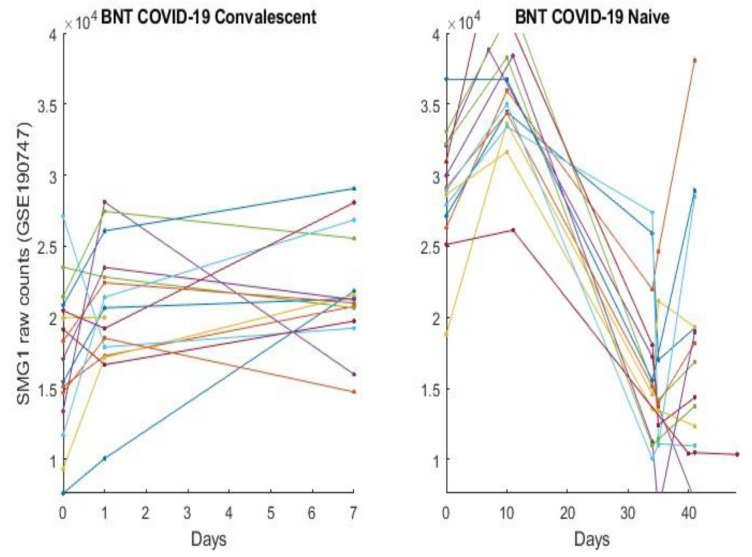
SMG1 expression responses to BNT162b2 vaccines: The left panel is for COVID-19-recovered octogenarians; the right panel is for COVID-19-naïve individuals. Each color line corresponds to one patient’s records.

**Figure 3:**
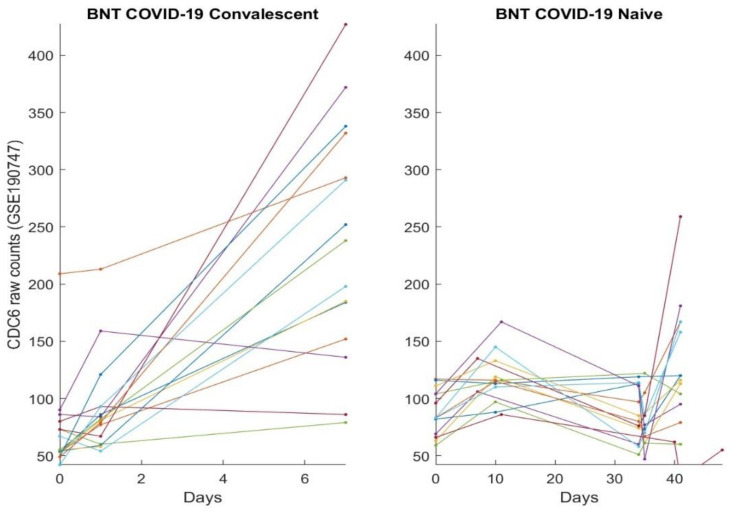
CDC6 expression responses to BNT162b2 vaccines: The left panel is for COVID-19-recovered octogenarians; the right panel is for COVID-19-naïve individuals. Each color line corresponds to one patient’s records.

**Figure 4:**
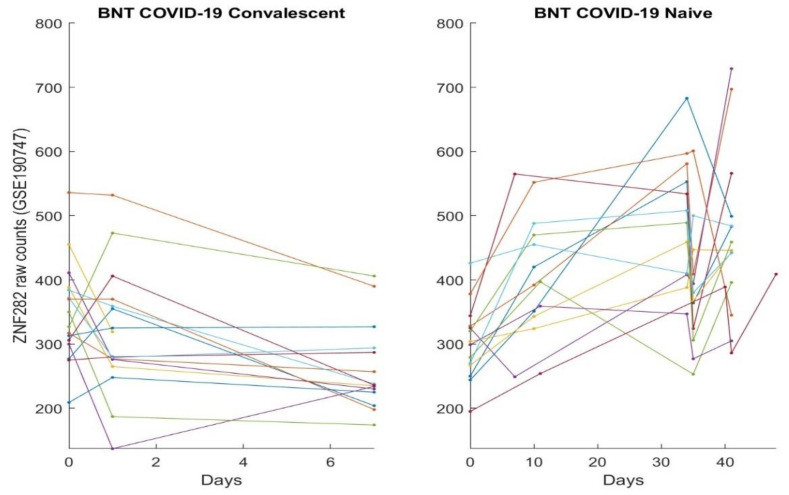
ZNF282 expression responses to BNT162b2 vaccines: The left panel is for COVID-19-recovered octogenarians; the right panel is for COVID-19-naïve individuals. Each color line corresponds to one patient’s records.

**Figure 5:**
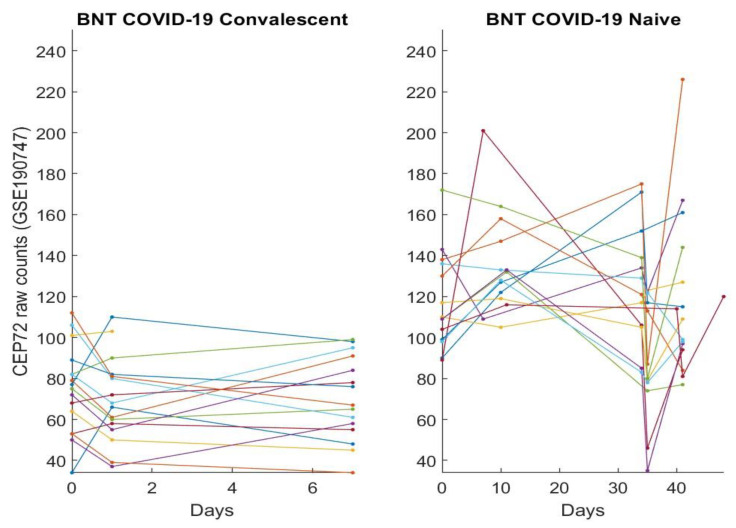
CEP72 expression responses to BNT162b2 vaccines: The left panel is for COVID-19-recovered octogenarians; the right panel is for COVID-19-naïve individuals. Each color line corresponds to one patient’s records.

**Figure 6:**
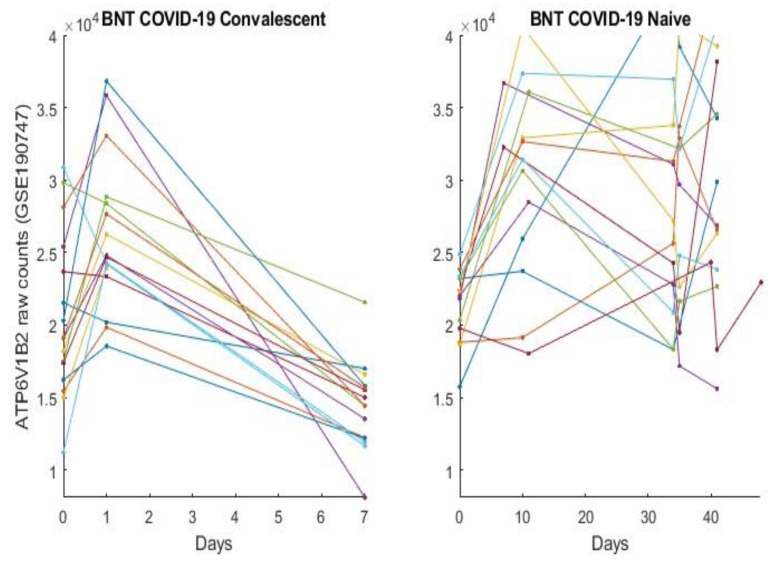
ATP6V1B2 expression responses to BNT162b2 vaccines: The left panel is for COVID-19-recovered octogenarians; the right panel is for COVID-19-naïve individuals. Each color line corresponds to one patient’s records.

**Figure 7:**
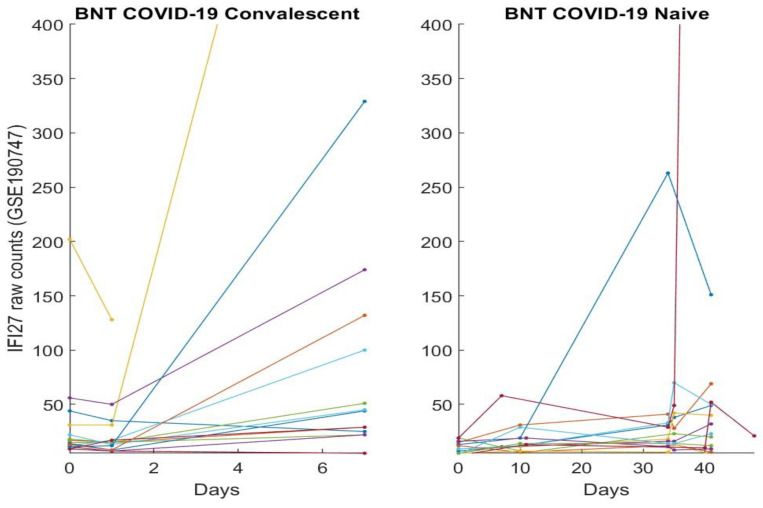
IFI27 expression responses to BNT162b2 vaccines: The left panel is for COVID-19-recovered octogenarians; the right panel is for COVID-19-naïve individuals. Each color line corresponds to one patient’s records.

In this section, we directly plot gene expression change responses to the BNT162b2 vaccine. Using the genes identified in our earlier work and the last section as COVID-19 biomarkers, in the subsequent figures, we plot MND1, SMG1, CDC6, ZNF282, CEP72, ATP6V1B2, IFI27 responses in Figure 1, Figure 2, Figure 3, Figure 4, Figure 5, Figure 6, and Figure 7, respectively. 

Figure 1, Figure 2, Figure 3, Figure 4, Figure 5, Figure 6 and Figure 7 clearly show that COVID-19-convalescent individuals and COVID-19-naïve individuals have entirely different BNT162b2 vaccine responses. Our earlier work [27] used a total of fourteen cohort studies (including different platforms, different ethics, different geographical regions, breakthrough infections, and Omicron variants) with 1481 samples to justify the results. So far, we have not seen any other research in the literature with nearly perfect performance. With such comprehensive studies and conclusive outcomes, it may be safe to say that the identified genes in our earlier work are representative, and the gene–gene interaction heterogeneity between SARS-CoV-2 and COVID-19 does exist. Using the results from our earlier work [26,27] and those in Section 3.1, we can see that the higher the expression values of ZNF282, CPE72, and ATP6V1B2, the lower the risk of an individual being COVID-19 positive; and the lower the expression values of MND1, CDC6, and IFI27, the lower the risk of an individual being COVID-19 positive. Clearly, COVID-19-naïve individuals have improved and expected vaccine responses, i.e., the BNT162b2 vaccine can help prevent SARS-CoV-2 infections for COVID-19-naïve individuals. 

Note that at time zero, except for SMG1 and CEP72, the two groups are comparable in terms of their expression values. However, COVID-19-convalescent octogenarians showed adverse effects with other genes, i.e., the BNT162b2 vaccine could increase the risk of breakthrough SARS-CoV-2 infections in this group of individuals. 

Figure 2 shows different patterns of SMG1 expression level changes. In our earlier work [25,26], we found that this mRNA gene can be either helpful or harmful depending on its combination effects with other genes. The right panel also shows that the vaccine can be either helpful or harmful depending on its effect time. 

### 3.3. Separability between COVID-19-Naïve Individuals and COVID-19-Convalescent Octogenarians Using the High-Performance Biomarkers

The figures in Section 3.2 showed the significant difference between two heterogeneous populations using reliable high-performance biomarkers. In this section, we use the seven genes to separate two BNT162b2 vaccine populations. We fit the max-linear competing logistic classifier model to the data and obtained the following Table 6 using only four genes: MND1, SMG1, CEP72, and APT6V1B2. 

In the table, sensitivity is for the COVID-19-naïve population, and specificity is for the COVID-19-convalescent population. We have an overall accuracy of 88.70%, with 89.71% sensitivity and 87.23% specificity. Such performance clearly shows that these two populations have very different responses to the BNT162b2 vaccine, which provides justifications for direct findings in Section 3.2.

## 4. Discussions

The graphical results in Section 3.2 were direct illustrations, without any statistical calculation and modeling, of gene expression level changes (of clinic information) over time. They show biological phenomena and patterns. Section 3.3 used only four genes to study the significant differences between two heterogeneous populations with an overall high performance of 88.70% accuracy. These results show that the genes used in this study contain basic genomic information, i.e., their responses to the BNT162b2 vaccine. The significant differences between responses from two heterogeneous populations are apparent and convincing. 

Our work to study the efficacy of vaccines is completely different from existing studies in the literature, which detected antibody responses, immune responses, durations of neutralizing antibody responses, immune boosting, etc. This paper, at the genomic level, used high-performance biomarkers to study genomic (gene) responses to the BNT162b2 vaccine in two heterogeneous populations. The chosen genes (biomarkers) led to 100% accuracy in our earlier work [26] and the new Table 3 in this paper. These biomarkers characterize the COVID-19 disease at the genomic level, the results are interpretable, and hence they provide genomic information on the disease’s pathological relationship to genes and their functional effects. Given that the knowledge of COVID-19 is still limited, it may be safe to say that knowing the genomic relationship between the disease and the high-performance and reliable biomarkers can lead to better vaccine practice and development and antiviral drug development than the current knowledge of SARS-CoV-2. The current vaccines have not evaluated the responses of the biomarker genes and their functional effects in their formulas. There is a potential that the vaccines can make the disease even worse as they can result in adverse effects on gene expressions and their joint functions with other genes. Our new results of the gene–gene interaction effects at the genomic level reveal a new direction for next-generation vaccine development, such that they can more efficiently prevent infection. Using the flu vaccine as an analog example, it has been reported that it is just 15% effective this year. One can say that this is due to new flu variants. However, it may be due to the intrinsic gene–gene interactions, and their functional effects have not been utilized in the vaccine formulas. 

Our earlier work [26] proved that the existence of COVID-19 genomic signature patterns with the single-digit number of genes determines the recurrence of COVID-19 disease; especially, recurrence (breakthrough infection) can occur in COVID-19-convalescent individuals with a higher probability, as these individuals’ COVID-19 genomic signature had been observed in their genetic system. Our undeniable results in Section 3.2 raise outstanding concerns about allowing COVID-19-convalescent octogenarians to take the BNT162b2 vaccine. A potential risk is that these individuals may suffer breakthrough infections. The logic is that these individuals may have adverse effects from vaccines and change the critical gene expression levels to higher/lower the threshold value of being healthy. 

A key question is when they can take a vaccine after recovering from a COVID-19 infection. 

Our earlier work [27] hypothesized that MND1 and CDC6 could be responsible for the virus (genetic segment) replication and mutation, and their combined effects can lead to new variants. However, ZNF282 can be hypothesized as a repair agent of SARS-CoV-2. Therefore, these genes demand further studies, aiming to make vaccines and antiviral drugs to lower or control the expression levels of MND1 and CDC6 but to boost the expression levels of ZNF282. In addition, given that ZNF282 is a zinc-figure protein gene, it is worth looking further into whether or not zinc supplements can be helpful. 

## Figures and Tables

**Table 1:** Performance of individual classifiers and combined max-competing classifiers using blood-sample data GSE157103 to classify hospitalized COVID-19 patients and other types of patients (as control) into their respective groups. CF-I, II, and III are three different classifiers. CFmax = max (CF-I, II, III) is the combined max-competing classifier. TPM stands for transcripts per million, and EC stands for expected counts. The numbers are fitted coefficient values.

Classifiers	Intercept	ABCB6	KIAA1614	MND1	RIPK3	SMG1	Accuracy	Sensitivity	Specificity
CF-I (TPM)	−0.3303		3.4153	0.2177		−0.1248	69.84%	62%	100%
CF-II (TPM)	−0.7378	−0.462		0.9093		0.0654	80.16%	75%	100%
CF-III (TPM)	6.9282				−0.3921		34.13%	17%	100%
CFmax							100%	100%	100%
CF-I (EC)	−0.7877		0.0351	0.0181		−0.0008	59.52%	49%	100%
CF-II (EC)	−4.6701	−0.0408		0.2134		0.0014	73.02%	66%	100%
CF-III (EC)	3.1584				−0.0042		58.73%	48%	100%
CFmax							100%	100%	100%

In the table, the classifier CF-I(TPM) is defined as −0.3303 + 3.4152 × KIAA1614 + 0.2177 × MND1−0.1248 × SMG1, using 0.5 as the threshold in computing risk probability in the logistic regression function. Other classifiers are defined similarly. All mathematical equations, algorithms, and interpretations are referred to by Zhang [26].

**Table 2:** Performance of individual classifiers and combined max-competing classifiers using blood-sample data GSE152418 to classify COVID-19-infected and healthy controls into their respective groups. The meaning of CF-i is the same as those in Table 1. Raw stands for raw counts.

Classifiers	Intercept	ABCB6	KIAA1614	MND1	RIPK3	SMG1	Accuracy	Sensitivity	Specificity
CF-I (Raw)	9.0357		−0.0611	0.1628		−0.0089	97.06%	94.12%	100%
CF-II (Raw)	9.2613	−0.2191		0.1963		–0.0081	97.06%	94.12%	100%
CFmax							100%	100%	100%

**Table 3:** Performance of individual classifiers and combined max-competing classifiers using blood-sample data GSE201530 to classify COVID-19-infected and healthy controls into their respective groups. The meaning of CF-i is the same as those in Table 1. Raw stands for raw counts.

Classifiers	Intercept	ABCB6	MND1	RIPK3	SMG1	CDC6	ZNF282	CEP72	Accuracy	Sensitivity	Specificity
CF1 (Raw)	−1.6909				0.0001	2.0352	−0.6842		50.91%	42.55%	100%
CF2 (Raw)	−7.5469	−0.9264	5.8238					1.9166	80%	76.60%	100%
CF3 (Raw)	1.466	0.4688	−1.4305	−0.0862					20%	6.38%	100%
CF4 (Raw)	3.0641	−0.8549			0.0001		0.6613		70.91%	65.96%	100%
CFmax									100%	100%	100%

**Table 4:** Characteristics of the top-performing five-gene classifier using the data from [35] and NP/OP swab PCR-sample data to classify COVID-19 patients and other viral ARIs and non-viral patients into their respective groups.

Classifier	Intercept	ATP6V1B2	IFI27	BTN3A1	SERTAD4	EPSTI1	Accuracy	Sensitivity	Specificity
CF1	9.193	−1.8935	1.5774		−4.3303		87.61%	81.72%	91.49%
CF2	−7.2786	−5.2993		3.2572		2.34	86.32%	76.34%	92.91%
CFmax							91.88%	94.62%	90.07%

In addition to the NP/OP swab PCR-sample data in Table 4 [35], we studied another NP/OP swab PCR-sample data GSE152075 to obtain the following new Table 5.

**Table 5:** Characteristics of the three-gene classifier using the NP/OP swab PCR-sample data GSE152075 (log(Raw + 1)) to classify COVID-19-confirmed and COVID-19-negative data into their respective groups.

Classifier	Intercept	ATP6V1B2	SERTAD4	EPSTI1	Accuracy	Sensitivity	Specificity
CF1	−10.9845	−3.2959	−0.4205	7.6279	83.47%	83.49%	83.33%

**Table 6:** Performance of individual classifiers and combined max-competing classifiers using blood-sample data GSE190747 to classify BNT162b2 COVID-19-convalescent and SARS-CoV-2-naïve individuals into their respective groups.

Classifiers	Intercept	MND1	SMG1	CEP72	APT6V1B2	Accuracy	Sensitivity	Specificity
CF-I	−7.2068		−0.0001	0.0761	0.0001	85.22%	82.35%	89.36%
CF-II	−8.0074	−0.0251		0.0663	0.0001	83.48%	80.88%	87.23%
CFmax						88.70%	89.71%	87.23%

## Data Availability

The datasets are publicly available. The data links are stated in the section Data Description.

## Supplementary Materials

Real data and computer outputs are in a supplementary file available online: https://pages.stat.wisc.edu/~zjz/VaccineBH.zip. The results presented in this paper are all verifiable by simply checking the Excel sheets and formulas in the file.

## Limitation Statements

The data used in this study do not include wide-range age groups, gender groups, different ethics, and different types of vaccines. The sample size is just 30. However, our earlier work studying genomic biomarker heterogeneity had a large sample size, and the identified genes should be representative. A recent long COVID-19 exploratory research [38] has a modest size of 99 people, which went very deep into granular aspects of T cells and the antibody response, and it is the foundation for much bigger studies, as commented on in [39]. Our research in this paper is also exploratory, it went very deep into the genomic level, and it is the foundation for much bigger studies, too.
